# Sensing the squeeze: nuclear mechanotransduction in health and disease

**DOI:** 10.1080/19491034.2024.2374854

**Published:** 2024-07-01

**Authors:** Luv Kishore Srivastava, Allen J. Ehrlicher

**Affiliations:** a Department of Bioengineering, McGill University, Montreal, Canada; b Department of Biomedical Engineering, McGill University, Montreal, Canada; c Department of Anatomy and Cell Biology, McGill University, Montreal, Canada; d Centre for Structural Biology, McGill University, Montreal, Canada; e Department of Mechanical Engineering, McGill University, Montreal, Canada; f Rosalind and Morris Goodman Cancer Institute, McGill University, Montreal, Canada

**Keywords:** Chromatin, lamin A/C, LINC complex, mechanotransduction, nuclear deformation

## Abstract

The nucleus not only is a repository for DNA but also a center of cellular and nuclear mechanotransduction. From nuclear deformation to the interplay between mechanosensing components and genetic control, the nucleus is poised at the nexus of mechanical forces and cellular function. Understanding the stresses acting on the nucleus, its mechanical properties, and their effects on gene expression is therefore crucial to appreciate its mechanosensitive function. In this review, we examine many elements of nuclear mechanotransduction, and discuss the repercussions on the health of cells and states of illness. By describing the processes that underlie nuclear mechanosensation and analyzing its effects on gene regulation, the review endeavors to open new avenues for studying nuclear mechanics in physiology and diseases.

## Introduction

Enduring constant stresses from both internal and external sources, the nucleus exhibits a mechanical resilience to counter these forces and maintain the integrity of the chromatin within. Consequently, its mechanical properties are of paramount significance, as underscored by the emergence of pathologies associated with abnormal nuclear mechanics such as cancers [1,2] and laminopathies [3,4]. Nuclear deformability emerges as a direct consequence of nuclear mechanics; exposed to similar forces, increased nuclear stiffness results in reduced nuclear deformation, whereas a softer nucleus exhibits greater deformation. The extent of nuclear deformation can directly [5] and indirectly [6] influence gene expression within the nucleus, potentially contributing to pathologies associated with abnormal nuclear mechanics [3,7,8].

This review article provides an analysis of the existing knowledge about various components within the nucleus that serve as mechanosensors. After a review of nuclear components and some of their biological roles, it delves into the various sources of mechanical forces that act on the nucleus. Moreover, it explores the complex ramifications of alterations in these components, specifically within the framework of disrupted mechanotransduction, with a focus on the subsequent changes in gene expression that are ascribed to nuclear deformation.

## Overview of the structure of the nucleus

**Figure 1.**
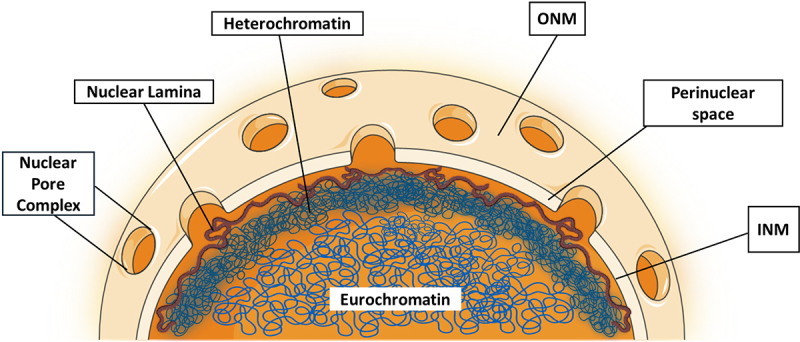
Ultrastructure illustration of the nucleus showing components like the nuclear envelope, nuclear lamina, heterochromatin and euchromatin.

In eukaryotic cells, the nucleus serves as the primary storage unit for genetic material, mainly the de-oxy ribonucleic acid (DNA) [9]. The nucleus is delimited by a double phospholipid membrane known as the nuclear envelope. The endoplasmic reticulum (ER) and the outer nuclear membrane (ONM), studded with ribosomes on its cytosolic surface, establish a tightly integrated membrane continuum [10]. The perinuclear space, located between the inner nuclear membrane (INM) & ONM typically measures approximately 30 to 50 nm in width [10]. The Nuclear pore complexes (NPCs) span the perinuclear space at sites of INM-ONM fusion, and serve as essential conduits for the trafficking of molecules and the transfer of information between the cytoplasm and the nucleus [11]. They facilitate the bidirectional exchange of macromolecules, regulating the passage of ions, proteins, and nucleic acids across the nuclear envelope, thus orchestrating fundamental cellular processes. The INM assumes a pivotal role in regulating gene expression by overseeing the organization and accessibility of chromatin, a combination of DNA and proteins inside the nucleus, thereby contributing significantly to the precise orchestration of genetic information [12]. Beneath the INM lies the nuclear scaffold, also referred to as the nuclear lamina, a mechanically rigid structure [13] as shown in Figure 1. It is composed of a variety of lamin protein such as lamin A,C,B1 and B2 [14] that cooperate to shield the inner chromatin, which is the condensed form of genomic DNA and can exist as either heterochromatin or euchromatin, as will be covered later in the review. By preserving cellular regulation and genetic control, nuclear lamins provide the nucleus with a mechanical rigidity that is essential for the normal physiology of the cell [15]. Without these, the genome is more vulnerable to outside forces acting on the nucleus [16,17].

## Mechanics of the nucleus

The Young’s modulus, which signifies material stiffness, of the cellular nucleus typically varies between 1 and 10 kPa [18,19], which is substantially higher compared to the estimated modulus of the cytoplasm (10 Pa) [20]. It is important to understand that the absolute modulus values can vary based on the type of cell and the testing methods used.

The modulus of the cellular nucleus may experience substantial fluctuations, with increases of up to 5-fold observed during cell division and 6-fold during stem cell development [21,22]. Notably, several cancer cell types are usually associated with a decrease in nuclear stiffness [1,23], with variations in the nuclear envelope architecture such as irregular and more deformed nuclei accounting for most of these abnormalities [24]. Conversely, conditions known as laminopathies, which result from mutations in nuclear lamins and related proteins, like Hutchison-Gilford Progeria syndrome [3,25] are associated with abnormal nuclear structure and increased nuclear stiffness. The nuclear lamina, which is mostly made up of nuclear lamins, primarily determines the mechanical properties of the nucleus along with the chromatin present inside the nucleus. Notably, chromatin is responsible for nuclear responses to small extensions, while lamin A/C levels control nuclear strain stiffening at large extensions [26].

The majority of eukaryotic cells employ a network of intermediate filament type V proteins known as lamins. These structural components can be broadly categorized into two groups: A-type and B-type. A-type lamins, encompassing lamin A and its splice variant lamin C (encoded by the *LMNA* gene) [27,28], primarily appear in differentiated cells, exhibiting limited expression during embryonic development [29]. In contrast, B-type lamins, including lamin B1 and lamin B2 (encoded by *LMNB1* and *LMNB2* genes, respectively) [30], display ubiquitous expression across diverse developmental stages [31]. This varied expression pattern shows that each lamin type has unique functions in different stages of development. Lamins have three domains: the head for inner nuclear membrane binding, the alpha-helical rod for structure, and the tail for chromatin attachment [32]. Their assembly at the nuclear membrane (NM) is tightly regulated through post-translational modifications, including cleavage and phosphorylation, orchestrated by a cascade of enzymatic reactions [33]. A-type lamins maintain nuclear shape and stability [7,34], whereas B-type lamins, essential from embryonic development to adulthood, play crucial roles in gene expression and cellular integrity [35,36]. Alterations in B-type lamins can lead to developmental defects and cellular abnormalities, evidenced by nuclear blebbing and gene regulation issues in lamin B1-deficient mice [31,37].

The nuclear lamina serves as a key determinant of nuclear stiffness mainly because of the contribution of lamin A/C. This crucial role of lamin A/C in nuclear mechanical properties has been extensively documented, with numerous studies demonstrating a direct link between reduced lamin A/C concentration and decreased nuclear stiffness [38–40]. Specifically, experiments involving substrate stretching reveal a notable 30% to 50% increase in nuclear deformation in cells lacking A/C compared to their wild-type counterparts [41]. Mounting evidence suggests a direct link between reduced lamin A/C expression and compromised nuclear mechanical integrity. Studies have demonstrated increased susceptibility to nuclear rupture under elevated nuclear stress in cells with low lamin A/C levels [42]. Magnetic bead microrheology experiments also indicate that nuclei originating from cells with diminished lamin A/C levels experience bead displacement 1.5–1.7 times greater than that observed in wild-type nuclei [40] illustrating the reduction of nuclear stiffness with lower lamin A/C expression. Indeed, nuclear stiffness and lamin A/C expression are directly related and thus lamin A/C expression can also regulate other physiological properties dependent on nuclear stiffness. For instance, increased lamin A/C expression stiffens the nucleus and blocks three-dimensional cell motion via 3 µm transwells, but it does not affect migration through larger pores [43]. This happens because increased lamin A/C expression leads to a much stiffer and rigid nucleus making it challenging for the nucleus to squeeze through small 3 µm pores. In addition to its role in overall nuclear stiffness, lamin A/C, in conjunction with chromatin, regulates local sub-micron nuclear stiffness, displaying significant spatial variations across the nucleus [38]. It is to be noted that in addition to lamin A/C, other forms of nuclear lamins have also been shown to maintain nucleocytoskeletal interactions and cellular mechanics [44].

Beyond the nuclear lamina, chromatin stands as another crucial determinant of nuclear mechanical properties [45]. This intricate network of DNA and proteins provides the structural foundation for chromosomes within the nucleus [46]. The accessibility of regulatory factors to DNA, governing gene expression, is tightly linked to chromatin condensation states [47]. Notably, chromatin organization, sculpted by the interplay of DNA, histone proteins, and other nuclear elements, orchestrates a plethora of cellular processes, including transcription, DNA replication, and DNA repair [48,49]. Moreover, chromatin exhibits remarkable adaptability, undergoing modifications such as histone modifications [50], chromatin remodeling [51], and DNA methylation [52]. These epigenetic changes exert profound influence on various biological functions, encompassing cell differentiation, DNA rearrangements, and chromosome stability [53,54]. Notably, histone modifications like acetylation [55], methylation [52], and phosphorylation [56] play a pivotal role in shaping chromatin dynamics and impacting gene expression. Additionally, enzyme-guided chromatin remodeling dictates DNA accessibility for transcription and repair. Within the nucleus, chromatin adopts two distinct forms: heterochromatin, the condensed and transcriptionally inactive form typically residing at the periphery [57], and euchromatin, the gene-rich and transcriptionally active form occupying the central region [58]. These forms are distinguished by their unique protein interactions and histone modifications, which govern gene silencing in heterochromatin and promoting gene activity in euchromatin.

It is imperative to acknowledge that lamins and chromatin are not autonomous components of the nucleus; rather, they are mechanically coupled [59]. Chromatin segments, referred to as lamina-associated domains (LADs), establish mechanical contact with the nuclear lamina, thus forming a link between the two components [60]. This has substantial importance, as chromatin detachment from the nuclear envelope results in a 20% increase in nuclear deformation [61]. The degree of heterochromatin, which is distinguished by its dense conformation in contrast to euchromatin, substantially influences the stiffness of the nucleus: the results of experimental stretching indicate that a 3-fold increase in chromatin compaction results in a corresponding 40% to 60% rise in nuclear stiffness [26]. On the other hand, chromatin that has experienced enzymatic digestion and resulted in a 2-to-3-fold increase in euchromatin confirmation by histone acetylation reduces nuclear stiffness by 30% to 50% for small, applied forces. The stiffness of the nucleus is approximately doubled when histone deacetylase (HDAC), an enzyme that facilitates chromatin compaction (heterochromatin) by eliminating acetyl groups from histone proteins, is overexpressed in HT-29 human colon cancer cells [26]. Conversely, the stiffness of wild-type HT-29 nuclei is increased when these cells are exposed to HDAC inhibitors such as valproic acid and trichostatin A which are responsible for inhibiting HDAC1, 2 &3. Chromatin can also be decondensed using a broad-spectrum histone methyltransferase inhibitor, such as 3-Deazaneplanocin-A or DZNep. The reduction in nuclear stiffness by approximately 40%, predominantly attributed to DZNep-induced epigenetic modifications in heterochromatin methylation (H3K9me3, H3K27me3, H4K79me3, and H4K20me3) [62], highlights the significance of chromatin condensation in regulating nuclear stiffness [26].

## Forces at play

The nucleus exhibits remarkable dynamic behavior, undergoing continuous morphological, positioning, and reorientation shifts during pivotal cellular processes like migration [63], differentiation [64], and cell division [65]. These crucial alterations are tightly coordinated by cytoskeletal connections that physically link the nucleus to the rest of the cell [66]. At the core of this intricate communication network lie specialized structures known as Linker of Nucleoskeleton and Cytoskeleton (LINC) complexes, playing a central role in anchoring the nucleus to and facilitating its dynamic interactions with the surrounding cytoskeleton [67].

These mechano-molecular bridges facilitate the bidirectional transmission of forces and biochemical signals, serving as critical communication conduits between these cellular compartments [68]. Multiple proteins form the LINC complexes, which connect the cytoskeleton to the nuclear interior [66].

Nesprins, a type of spectrin repeat-containing protein localized in the ONM as well as INM [69], constitute a fundamental element of LINC complexes when situated at the ONM [70]. These nesprins engage in interactions with proteins on the INM, specifically those harboring the SUN (Sad1p, UNC-84) domain, via their distinctive C-terminal KASH (Klarsicht, ANC1, Syne homology) domain [71]. The complex interaction between Nesprins and SUN/KASH is essential for determining the spatial relationship between the ONM and INM [72]. Beyond its established role in regulating molecular exchange, the nuclear envelope facilitates the transmission of mechanical and biochemical signals [5]. Disruption of this finely tuned process can have significant structural and functional consequences for the cell. Impaired communication between the cellular exterior and the nucleus compromises the cell’s ability to sense and respond to its environment, potentially leading to abnormal nuclear mechanotransduction [73,74] and a range of pathologies, including Dunnigan-type familial partial lipodystrophy [75], Werner syndrome [76], and Charcot-Marie-Tooth syndrome 2B [77].

Only the so-called ‘Giant’ isoforms of Nesprin 1 and Nesprin 2, known to interact with actin filaments and possess a transmembrane domain, engage with actin filaments and microtubules on the cytoplasmic side of the complex, facilitated by kinesins and dynein [78]. In addition, it is worth noting that there exist numerous tissue-specific isoforms of Nesprin 1 and Nesprin 2. These tissue-specific isoforms may exhibit distinct functionalities and localization patterns, contributing to the diverse roles of Nesprins in various cellular contexts [79]. Nesprin 3 binds with intermediate filaments via its docking interaction with plectin [80] and lastly Nesprin 4 assists in nuclear location inside specialized polarized epithelial cells through its interaction with kinesin 1 [81]. Aside from Nesprins, other KASH domain proteins include KASH5 (CCDC155) and KASH6 (Jaw1/LRMP). KASH5 is a protein specific to meiosis that links meiotic chromosomes to the cytoskeleton [82]. KASH6 is a distinct KASH protein found in the ER and ONM, with specialized expression in certain immune system cells [83].

Via the LINC complex, the mechanics outside the cell are transmitted to the nucleus which eventually determines its shape [84–86]. For instance, when a cell is cultured on a soft substrate, the mechanical forces it experiences from tension in the actin cytoskeleton, a network of protein filaments that helps maintain cell shape and structure, are small and evenly distributed. This is because soft substrates are deformed more when cells exert force on them [87,88]. As a result, cells on soft substrates do not need to generate as much contractile force to achieve the same level of substrate deformation leading to a less tense actin cytoskeleton and less force on the nucleus [89]. In this scenario, the nucleus typically assumes a more spherical shape [84]. On the other hand, when a cell is adhered to a stiff substrate, like a rigid plastic surface, the mechanical forces from actin tension exerted on the cell are much stronger and localized. This increased tension in the actin cytoskeleton results in the cell nucleus being subjected to larger forces, and greater deformation, particularly compression in the vertical dimension [90], causing the nucleus to adopt a more flat or elongated shape under these conditions [86]. This provides direct mechanical feedback between microenvironment mechanics and nuclear deformation and geometry, with profound implications for nuclear mechanotransduction.

## Nuclear mechanotransduction

Nuclear mechanotransduction refers to the process by which mechanical signals are converted into biochemical responses within the cell nucleus. Understanding how cells react to mechanical stimuli, like pressure or tension, and how these stimuli affect gene expression and cellular behavior, depends heavily on this intricate system, which encompasses a network of signaling pathways, structural proteins, and regulatory mechanisms dedicated to transducing and interpreting mechanical cues within the nucleus. This process requires the nuclear envelope and associated proteins to convey mechanical stresses from the outside of the cell to the genome. These interactions can change the shape and function of chromatin, which can impact gene expression. This mechanism is crucial for many physiological processes, including tissue growth, wound healing, and the response of cells to their physical environment [84,91]. It is also becoming increasingly apparent that abnormal mechanical signals originating from the nucleus may contribute to the development of certain clinical disorders, such as cancer, where impaired nuclear mechanotransduction plays an influential role [8,13,92]. For instance, research by Irianto et al. revealed that the disruption of lamin-A in cancer cells results in more pliable nuclei, increasing the invasiveness and migration of cancer cells [93]. Additionally, it is known that physical stimuli applied to the nucleus can change the translocation of several growth regulators associated with cancer, such as Yes-associated protein (YAP) [5,94], nuclear actin [95], and transcription factors linked to the retinoic acid receptor (RAR) [96], establishing a connection between aberrant nuclear mechanotransduction and cancer. Nuclear mechanotransduction regulation is a complex process due to the vast array of proteins involved, and the diverse ways forces can deform nuclear components with expression consequences.

Chromosome stretching can increase gene accessibility for the transcriptional machinery, providing a strong example of how force can directly affect gene regulation [97]. One study used magnetic beads attached to integrins on the cell surface to stretch a reporter transgene that was fluorescently labeled; transgene transcription started as soon as 15 minutes after stretching, illustrating how force can alter chromatin organization inside the nucleus resulting in a change in gene expression [98]. Thus, mechanoregulation of 3D nuclear organization can directly impact nuclear mechanotransduction.

Recent studies, utilizing fluorescence in situ hybridization (FISH) on cells adhered to micropatterned substrates, have revealed a direct relationship between nuclear mechanics and chromosomal position [99–101]. While fibroblasts on high aspect ratio rectangular substrates have flattened nuclei and align chromosomes perpendicular to the major axis, transcription was shown to be higher on circular surfaces with spherical nuclei and perpendicular chromosome alignment [100]. These changes demonstrate the impact of mechanical constraints on gene expression patterns, chromosomal organization, and nuclear morphology.

Research using techniques like FISH has shown that some gene clusters become active when they are close together in space [102]. For instance, genes like the Hox gene cluster tend to be physically grouped. This clustering can be seen in experiments like chromatin conformation capture assays and chromatin immunoprecipitation followed by sequencing [103]. Even when cells are stimulated, certain genes, like TNFα target genes, remain close together [104]. Disrupting these gene neighborhoods can affect the expression of genes controlled by NF-κB [105]. This is significant in the context of nuclear mechanotransduction, where mechanical forces affect gene expression, as it implies a connection between alterations in the mechanical characteristics of the nucleus and how genes cluster together. The order in which genes cluster in space can also impact how they work together [106]. This observation helps us understand how the genome’s spatial organization is influenced by mechanical and chemical signals, which in turn affects how genes are regulated for specific cell functions and nuclear mechanotransduction pathways.

In addition to gene localization, force application also causes the repositioning of other protein components of the cytoplasm, nucleus, and nucleoplasm which affects nuclear mechanotransduction [107]. One such protein is the NM protein Emerin, which is essential for controlling nuclear mechanics and via its ability to transmit polarity from the cytoskeleton to the nucleus can regulate mechanotransduction [108]. This role is particularly clear from the aberrant nuclear shapes, lesions, and changes in gene transcription that are seen in cells with mutated or absent Emerin [7,34], as well as from its link to Emery-Dreifuss muscular dystrophy (EDMD) in humans [109]. Although Emerin’s direct impact on nuclear stiffness is not well understood, one theory suggests that it may do so through interactions with lamin A [110]. Emerin directly binds to and stabilizes actin filaments by capping their pointed ends in vitro. While it interacts with various proteins such as chromatin, nuclear actin, lamins, nesprins, and Barrier-to-Autointegration factor [111], the significance of these interactions for its role in actin polymerization is still debated in the field and more research is warranted. Emerin is more mobile migrating to peripheral ER [112] and less INM localized in certain situations, such as when Lamin A/C is deficient [113], although the expression of Lamin A proteins harboring EDMD-associated mutations can result in Emerin mislocalization [114]. Emerin’s anchoring via its LEM (LAP2, emerin, MAN1) domain to the nuclear lamina and chromatin impacts its retention at the INM and turnover. While the underlying mechanism remains unclear, mechanical strain can redistribute Emerin from the INM to the ONM [115]. This change has the potential to either enhance or hinder the polymerization of cytoplasmic actin, resulting in the reduction of nuclear globular actin (G-actin), which serves as a transcriptional coactivator and nuclear export factor. As a result, Emerin’s redistribution because of mechanical strain may influence transcription factor nuclear trafficking and gene expression.

It is now evident that a wide range of factors, such as those governing nuclear stiffness, extranuclear and intranuclear forces on the nucleus, and matrix stiffness, can regulate nuclear mechanotransduction. However, it is interesting to note that many of these factors converge and influence nuclear deformation, which appears to be a crucial factor influencing nuclear mechanotransduction.

## Nuclear deformation and nuclear mechanotransduction

Deciphering the intricacies of nuclear mechanotransduction hinges on a thorough understanding of nuclear deformation, a phenomenon governed by both the intrinsic mechanical properties of the nucleus and the applied mechanical forces [116–118]. Specifically, when comparing two nuclei with similar mechanical properties, the one subjected to greater stress will undergo more deformation. On the other hand, a nucleus with lower stiffness, when exposed to comparable stress, will deform more than its stiffer counterpart [41]. Given the nucleus’ central role in orchestrating cellular functions, nuclear deformation can trigger a cascade of short- and long-term effects. These encompass alterations in genome regulatory mechanisms [6], modifications in nucleo-cytosolic transport dynamics [94,107], and vulnerabilities in nuclear envelope integrity [3], ultimately leading to dysregulated mechanotransduction [118].

Nuclear deformation holds the potential to impact nuclear mechanotransduction both directly and indirectly [118]. The direct influence stems from local mechanical stresses transmitted to the LINC complex through various pathways, including the actin cytoskeleton, integrins, and epigenetic modifications in chromatin, such as H3K9me3 demethylation [6]. These modifications, induced by mechanical stresses, can modify transcriptional activity within the nucleus [100]. Nevertheless, nuclear deformation can also impact nuclear mechanotransduction indirectly by controlling the movement of transcription factors such as YAP [5,94,107]. When located within the nucleus, YAP can initiate the transcription of different genes that play a role in diverse cellular processes such as proliferation [119,120], differentiation [121], and migration [122,123].

Localized mechanical stresses, such as those resulting from actin fiber-based indentation of the nucleus, can induce significant deformation of the nuclear envelope [86,87]. This process may lead to the reversible formation of heterochromatin [6]. Moreover, nuclear deformation may increase the activities of histone methylases and HDACs. Consequently, markers of heterochromatin, such as H3K9me3 and H3K27me3, increase, facilitating cell migration through mechanisms that are not fully understood [6].

Together with localized changes in chromatin organization and structure, dynamic nucleus deformations may serve as triggers for spatiotemporal genomic reconfiguration. The development of rod photoreceptors from their progenitor cells in mice illustrates this: it is known that in their precursor stage, rod photoreceptors have normal nuclear architecture, but during differentiation, they acquire an inverted condition with reduced peripheral and increased central heterochromatin and chromocenters [124]. The dynamic nuclear deformation that takes place during differentiation has been linked to the movement of heterochromatin and chromocentres [125].

Emerging evidence highlights the profound impact of mechanical stimuli on epigenetic regulation, ultimately influencing gene expression and cell fate [126,127]. Human mesenchymal stem cells (hMSCs), for instance, exhibit increased nuclear membrane tension and histone acetylation levels in response to stiff extracellular matrices (ECMs). This phenomenon, attributed to the deactivation of HDACs [128], ultimately steers hMSC differentiation toward osteocytes [128,129]. Conversely, disruption of LINC complexes leads to upregulated HDACs, potentially decreasing nuclear membrane tension and hindering osteogenic differentiation [128]. Similarly, sustained fibroblast differentiation into myofibroblasts necessitates both increased HDAC activity and nuclear compression arising from cytoskeletal stresses mediated by LINC complexes [130].

Moreover, mechanotransduction can be influenced indirectly by nuclear deformation; an increase in membrane tension caused by nuclear deformation facilitates the translocation of several transcription factors that are accountable for the transcription of diverse genes via the opening of nuclear pore complexes [5].

The NPC, when examined structurally, is an intricate assemblage of protein components, consisting of around 30 unique nucleoporins [131]. The protein complex is capable of being divided into three discrete compartments: the central transporter, the cytoplasmic ring, and the nuclear ring. Multiple nucleoporins are encased in the cytoplasmic ring, which also serves to secure the NPC to the cytoplasmic side of the nuclear membrane [11]. The nuclear ring, which is composed of several nucleoporins, serves to affix the NPC to the nucleoplasmic terminus of the nuclear membrane. The central transporter is composed of a multitude of nucleoporins that provide a pathway for the passage of molecules [132].

NPCs are crucial for regulating the passage of vital molecules from the nucleus to the cytoplasm. Although smaller substances, like ions and metabolites, are capable of diffusing through NPCs without hindrance, specific transport pathways are required for bigger molecules like proteins (>40kDa or 39 nm in diameter) and RNA [133,134]. As a result, several variables influence the movement of molecules via NPCs, such as the conformation and dimensions of the molecules, the existence of transport signals, and the interaction between transport receptors and transport signals [135]. Passive diffusion, which permits the unguided flow of smaller molecules across the NPC, and active transport, a selective mechanism requiring the engagement of transport receptors for molecules greater than about 40kDa, are the two main classifications of nuclear shuttling [136]. Active diffusion is primarily controlled without mechanical influence, but passive diffusion may be influenced by mechanical forces acting on the nucleus. The absence of a motor in nucleoporins means that changes in NPC diameter due to mechanical stress on the nucleus indicate that nuclear membrane tension controls the NPC diameter [137,138], allowing for the passive diffusion of specific components crucial for the cell’s normal physiology [5].

The precise regulation of protein and RNA importation into the nucleus is carried out by nuclear transport receptors (NTRs) and nuclear localization signals (NLSs) [139,140]. NTRs identify NLSs, which are composed of short amino acid sequences, and subsequently attach to the cargo to aid in its transit through the NPC. Numerous NTRs, including transportin, importin alpha/beta, and karyopherin beta 2, have been found. Additionally, various RNA-binding proteins, including transportin-SR (which is abundant in serine/arginine) and heterogeneous nuclear ribonucleoproteins, regulate RNA importation into the nucleus [141,142].

On the other hand, the exit of RNA and proteins from the nucleus is regulated by nuclear export receptors and nuclear export signals (NESs) [143]. NESs are concise sequences of amino acids that export receptors recognize, allowing them to attach to the cargo and facilitate its passage through the NPC. To aid this process, many export receptors, including exportin 1 and chromosomal region maintenance (CRM1) [144], have been found. Furthermore, the process of RNA expulsion from the nucleus is governed by specific RNA-binding proteins, namely exportin-t and Mex67-Mtr2 [140].

Apart from NPCs, other types of stress, such as osmotic stress, have also been demonstrated to trigger a series of phosphorylation or dephosphorylation processes. These reactions can impact the translocation of specific proteins such as YAP and Nuclear factor of activated T cells 5 (NFAT5) [145], rendering their nuclear translocation independent of NPCs regulation [146,147].

**Figure 2.**
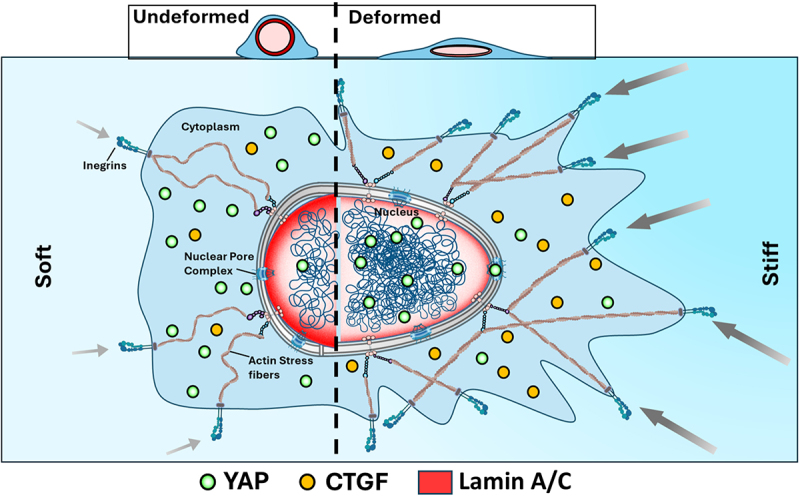
A schematic illustration that irrespective of the mechanism of nuclear deformation, leads to elevated YAP nuclear translocation and enhanced expression of downstream proteins such as connective tissue growth factor (CTGF).

YAP and transcriptional co-activator with PDZ-binding motif (TAZ), key transcriptional coactivators, exert widespread influence over various biological processes [148]. Their critical roles encompass diverse cellular activities such as proliferation [120,149], migration [123], and stem cell differentiation [121]. The aforementioned factors induce the nuclear envelope to undergo stretching, which may potentially facilitate the opening of NPCs and augment the import of YAP [5,150]. Conversely, in the process of myoblast differentiation into myotubes, nuclear elongation stimulates the nuclear export of YAP directing cell differentiation [151]. Recent research has also linked the nuclear export of YAP to modifications in the curvature of the substrate, which in turn causes nuclear deformations: when positioned on convex surfaces, nuclei grow flatter, YAP is more abundant in the nucleus, and chromatin becomes less condensed. In contrast, chromatin is more densely packed in nuclei situated on concave surfaces, which experience substantial elongation, where YAP is mostly localized in the cytoplasm and inactive [152]. The results of this study provide further evidence in favor of the notion that nuclear deformations impact YAP/TAZ nuclear-cytoplasmic transport, highlighting the importance of cytoskeletal and mechanical forces acting on nuclei. A recent study has shown that nuclear deformation through external pressure or cell contractility leads to similar elevated nuclear localization of YAP [94], suggesting that external forces achieve the same effects as intracellular forces. A schematic representation of how nuclear mechanotransduction is affected by nuclear deformation via YAP translocation has been shown in Figure 2.

There are several lines of evidence indicating that comparable outcomes may be witnessed when nuclear deformations are induced via external pressures [146] or increased cell density [153], but the mechanical characteristics of the ECM remain unchanged. Nonetheless, the exact mechanisms that regulate the intracellular localization of YAP in reaction to alterations in the volume and shape of the nucleus, together with their correlation with established regulators of YAP nuclear translocation, are still not completely understood.

In addition to controlling mechanotransduction through mechanosensitive proteins, nuclear deformation also controls the localization of other components, such as calcium ions, which are crucial for cellular signaling. This occurs frequently in tandem with processes like NM stretching. Cellular deformation elevates cytoplasmic and/or nucleoplasmic Ca^2^ + via mechanosensitive channels on the plasma membrane (PM) or in the ER, potentially activated by NM stretching due to ER-ONM continuity [91,154]. Lamin A/C depletion or Lamin B receptor overexpression disrupts NM properties, inhibiting Ca^2^ + transients in HeLa cells [155], while Lamin A/C overexpression increases Ca^2^ + release via Piezo1 in fibrosarcoma cells [156]. Cellular deformation has also been shown to bring ER and PM into spatial proximity, aiding Ca^2^ + influx via stromal interaction molecule-Orai mechanisms [157].

## Conclusion and future perspective

This review highlights the functions that nuclear mechanics and mechanotransduction fulfill in cell biology, while offering an overview of this field. Growing knowledge of the complex mechanisms governing the sense and transmission of mechanical forces within the nucleus of a cell indicates that these mechanisms have enormous potential to influence medicine in the future and demonstrate the nucleus’s ability to sense external forces and adapt by changing the cell’s genetic expression.

Because of its interaction with the chromatin and other mechanisms involving the translocation of mechanosensitive regulators, it is now clear that the nucleus is more than just a repository for genomic DNA; it is also able to respond to a variety of extranuclear and intranuclear mechanical factors that impact gene expression. Each of these components works in coordination; when they do not, a host of pathologies arise, ranging from Hutchison-Gilford Progeria Syndrome to cancer. Thus, a correlation between nuclear mechanics and how it may contribute to various disorders must be explored in future research.

The review also identifies nuclear deformation as a fundamental component driving nuclear mechanotransduction, which is corroborated by recent discoveries [5,90,107]. However, the focus of previous research has been on bulk nuclear deformation. Because of its heterogeneous distribution over the nuclear envelope, lamin A/C has previously been shown to contribute to variable local nuclear stiffness and deformation in addition to controlling bulk nuclear deformation [38]. Thus, to better comprehend the translocation of mechanosensitive regulators via NPCs opening and closure, attention must be directed toward the spatiotemporal mechanical features of the nucleus as well.

The nucleus functions closely with other cellular components, making it challenging to determine how the nucleus and other components contribute to mechanotransduction. Furthermore, the translocation of several mechanosensitive regulators can be regulated by both biological and mechanical pathways. For example, the mobilization of YAP is controlled by biological pathways like the Hippo pathway [158] in addition to the mechanical regulation discussed earlier [5,94,107], challenging distinguishing between the biological and mechanical contributions.

Looking ahead, the future of nuclear mechanotransduction research holds exciting prospects. One of the most promising areas of development is the translation of these insights into clinical applications. Understanding how mechanical forces influence cellular behavior opens doors to innovative therapies for various diseases, including cancer, cardiovascular disorders, and musculoskeletal conditions. Harnessing nuclear mechanotransduction may enable us to develop targeted interventions that can modulate nuclear mechanics, correcting cellular responses to improve patient outcomes.

Quantitative biophysics, which emphasizes quantifiable measures and physical principles, provides a powerful lens to study nuclear mechanotransduction. It uses fluorescence resonance energy transfer assays, atomic force microscopy, and live-cell imaging to directly monitor and analyze mechanical stresses on the nucleus and better understand how deformation affects NPCs, chromatin structure, and nuclear lamins.

Furthermore, quantitative biophysics delves into the molecular details of mechanotransduction. It enables the quantification of post-translational modifications and protein-protein interactions that govern the movement of molecules within the nucleus upon mechanical stimulation. This approach sheds light on the complex signaling networks at play, revealing how cytoskeletal forces, ECM stiffness, and nuclear envelope proteins dynamically interact during mechanotransduction.

This rich understanding transcends basic scientific curiosity, holding significant implications for various physiological processes and pathologies. Tissue development, cancer progression, and regenerative medicine methods are just a few areas where knowledge of nuclear mechanotransduction can prove invaluable. Additionally, by mapping the pertinent signaling networks, quantitative biophysics helps researchers identify critical regulatory nodes and pathways that respond to mechanical stress. This better understanding of how the nucleus transmits and processes mechanical stimuli paves the way for more accurate predictions of cellular responses and, ultimately, the development of customized treatments tailored to the needs of patients.

## Data Availability

Data sharing is not applicable to this article as no new data was created or analyzed in this study.
